# Lomustine Nanoparticles Enable Both Bone Marrow Sparing and High Brain Drug Levels – A Strategy for Brain Cancer Treatments

**DOI:** 10.1007/s11095-016-1872-x

**Published:** 2016-02-22

**Authors:** Funmilola A. Fisusi, Adeline Siew, Kar Wai Chooi, Omotunde Okubanjo, Natalie Garrett, Katerina Lalatsa, Dolores Serrano, Ian Summers, Julian Moger, Paul Stapleton, Ronit Satchi-Fainaro, Andreas G Schätzlein, Ijeoma F. Uchegbu

**Affiliations:** UCL School of Pharmacy, University College London, 29-39 Brunswick Square, London, WC1N 1AX UK; School of Physics, University of Exeter, Exeter, EX4 4QL UK; Department of Physiology and Pharmacology, Sackler School of Medicine, Tel Aviv University, Tel Aviv, 69978 Israel; Nanomerics Ltd. Euro House, 1394 High Road, London, N20 9YZ UK

**Keywords:** glioblastoma multiforme, lomustine, molecular envelope technology (MET), myelosuppression, nanoparticles

## Abstract

**Purpose:**

The blood brain barrier compromises glioblastoma chemotherapy. However high blood concentrations of lipophilic, alkylating drugs result in brain uptake, but cause myelosuppression. We hypothesised that nanoparticles could achieve therapeutic brain concentrations without dose-limiting myelosuppression.

**Methods:**

Mice were dosed with either intravenous lomustine Molecular Envelope Technology (MET) nanoparticles (13 mg kg^−1^) or ethanolic lomustine (6.5 mg kg^−1^) and tissues analysed. Efficacy was assessed in an orthotopic U-87 MG glioblastoma model, following intravenous MET lomustine (daily 13 mg kg^−1^) or ethanolic lomustine (daily 1.2 mg kg^−1^ - the highest repeated dose possible). Myelosuppression and MET particle macrophage uptake were also investigated.

**Results:**

The MET formulation resulted in modest brain targeting (brain/ bone AUC_0-4h_ ratios for MET and ethanolic lomustine = 0.90 and 0.53 respectively and brain/ liver AUC_0-4h_ ratios for MET and ethanolic lomustine = 0.24 and 0.15 respectively). The MET formulation significantly increased mice (U-87 MG tumours) survival times; with MET lomustine, ethanolic lomustine and untreated mean survival times of 33.2, 22.5 and 21.3 days respectively and there were no material treatment-related differences in blood and femoral cell counts. Macrophage uptake is slower for MET nanoparticles than for liposomes.

**Conclusions:**

Particulate drug formulations improved brain tumour therapy without major bone marrow toxicity.

## Introduction

Glioblastoma multiforme (GBM) is the most frequently occurring primary brain tumour in adults (accounting for 65% of primary brain tumours) and is the most aggressive, incurable malignancy of the central nervous system (1). GBM continues to be associated with poor prognosis, in spite of more recent therapeutic advances, with less than a quarter of patients surviving for 2 years after diagnosis (1–3). The median survival time is 14 months and only a very small percentage (3–5%) of patients survive for more than 3 years (4). As for most cancers, there is no actual cure for GBM.

GBM is characterized by uncontrolled cellular proliferation, diffuse infiltration and significant angiogenesis (5) and it is initially diagnosed using magnetic resonance imaging (MRI), although image interpretation is sometimes problematic (6). Once diagnosed GBM is treated by surgical resection followed by radiotherapy and chemotherapy, however, for some tumours there is no acceptable treatment (7,8). Chemotherapy, when indicated, is very challenging due to the heterogeneous and infiltrating nature of tumours and therapeutic agents being unable to access the tumour site; the latter due to the blood brain barrier (5,9,10). Therefore, methods to increase the localisation of chemotherapeutics to the intracranial tumour site are needed. Nitrosoureas such as carmustine and lomustine and other alkylating agents, *e.g.* temozolomide have been employed in GBM chemotherapy, however dose limiting toxicities, such as myelosuppression, limit the effectiveness of these drugs (11–14).

It is thus clear that increasing the drug levels at the tumour site while reducing drug levels in the bone marrow represent a significant challenge for the chemotherapy of GBM. One way of achieving high brain concentrations would be to increase the blood concentration of the drug *via* a dose intensification regimen. In the case of a lipophilic drug, we hypothesise that nanoparticles may allow high drug concentrations to be administered (dose intensification) such that high blood concentrations, and thus high brain tumour levels, of these drugs are achieved, without delivering high doses to the bone marrow. Dose intensification has been attempted in patients but requires autologous peripheral blood stem cell (PBSC) support *via* PBSC infusions (15).

We have chosen to test this bone marrow avoidance hypothesis with a class of nanoparticles which are known to evade liver capture on intravenous injection (16). These Molecular Envelope Technology (MET) nanoparticles are constructed from N-palmitoyl-N-monomethyl-N,N-dimethyl-N,N,N-trimethyl-6-O-glycol chitosan, a self-assembling polymer amphiphile (17). Drug loaded MET formulations were prepared and their biodistribution and pharmacodynamics/ toxic effects studied. We sought to provide a mechanistic explanation by also studying macrophage uptake of the MET particles.

## Materials and Methods

### Materials

All materials were obtained from Sigma Aldrich Corporation, MO, USA, unless otherwise stated. All solvents were obtained from Fisher Scientific, UK, Loughborough, United Kingdom.

### Cell Culture

U-87 MG human glioblastoma cell line was purchased from American Type Culture Collection (ATCC® HTB-14™; ATCC, Manassas, VA, USA). U-87 MG cells were grown in Minimum Essential Medium (Life Technologies, Paisley, UK) supplemented with FBS (10% ^v^/_v_; Labtech International Ltd, Uckfield, East Sussex, UK); sodium pyruvate (1 mM; Life Technologies) and L-Glutamine (2 mM; Life Technologies). Cells were maintained in culture (37°C in 5% CO_2_) for up to 14 days (splitting every 2–3 days when 75–80% confluence was reached in the 75 cm^2^ tissue culture flask) before they were used for tumour implantation.

### Synthesis of the MET Polymer

The MET polymer was synthesised as previously described (17). The characteristics of the MET polymer are shown in Table I.

**Table I:** MET Polymer Characteristics

Batch number	Mole% palmitoyl groups	Mole% quaternary ammonium groups	Molecular weight		
			Mw (kDa)	Mn (kDa)	Polydispersity
GCPQOO28072009	26.2	11.1	*	*	*
GCPQOO18082009	26.6	6.2	*	*	*
GCPQOO28112009	22	8	*	*	*
GCPQSR11112011	21.9	12.4	8.4	7.8	1.1
GCPQFF18042012	23.7	13.2	9.5	8.6	1.1
GCPQFF20022013	19.1	13.5	9.3	7.8	1.2
GCPQFF26032013	23.4	14.4	10.2	6.5	1.6

* = The molecular weight of these polymers is in the region of 9–10 kDa from other studies (17,18)

### Preparation of MET Lomustine Nanoparticles

The nanoparticle formulation was prepared by probe sonicating lomustine (2 mg ml^−1^), MET polymer (20 mg ml^−1^) soybean oil (10 mg ml^−1^) and polysorbate 80 (5 mg ml^−1^) in dextrose solution (5% ^w^/_v_) on ice for 30 minutes (MSE Sonipreo 150, MSE UK, with the instrument set at 50% of its maximum output). Each time 26 ml of the MET formulation was prepared. The formulation was filtered (0.22 μm) and the amount of lomustine encapsulated was measured by HPLC. To achieve high drug concentrations, the formulation from above (26 ml) was lyophilised and reconstituted to 13 ml using double deionised water (Millipore Water Purification System, EMD Millipore Corporation, Merck KGaA, Darmstadt, Germany). Formulations were analysed by HPLC using an analytical C_18_ derivatised silica gel based (Onyx Monolithic: 5 μm; 100 x 4.6 mm; Phenomenex®, UK) column using an Agilent (Agilent Technologies 1200 Series) HPLC system.

Formulations were sized using a Malvern Nanosizer (Malvern Instruments, Malvern, UK) at a temperature of 25°C and data analysed using the Contin method of analysis.

Formulations were imaged using transmission electron microscopy (TEM). For TEM imaging, a drop of the formulation was placed on a formvar/carbon coated grid and excess sample was blotted off on a filter paper (Whatman No 1). The samples were then negatively stained (uranyl acetate 1% ^w^/_v_) and left for 1–2 minutes to air dry. Subsequently, images were captured on the TE microscope using an AMT digital camera (5 mega pixels; AMT Deben, UK Ltd).

The stability of the dried lomustine nanoparticles was studied over a 7-day period, with formulations stored at room temperature and reconstituted periodically to produce a 1 mg ml^−1^ lomustine formulation, the drug content analysed and the particle size measured as described above.

### Preparation of the Lomustine Ethanolic Formulation

The ethanolic lomustine formulation administered to male CD-1 mice as control in the pharmacokinetic studies was prepared as follows: 5 μl of polysorbate 80 was transferred into a glass vial containing 2 mg lomustine. The vial was vortexed for 1 minute and 895 μl of 5% ^w^/_v_ dextrose solution was added to it. The vial was vortexed for another 1 minute. It was then sonicated on ice for 30 minutes as described above. 100 μl of 10% ^v^/_v_ ethanol was added to the vial containing the lomustine, polysorbate 80 and 5% ^w^/_v_ dextrose solution and was vortexed for 1 minute. The content of the vial was filtered through a 0.22 μm syringe filter (Millipore).

The ethanolic lomustine formulation administered as control in the pharmacodynamics and toxicity studies was prepared by vortexing lomustine (2 mg) in absolute ethanol (100 μl) with polysorbate 80 (5 mg ml^−1^) in 5% ^w^/_v_ dextrose solution (final ethanol concentration of 10% ^v^/_v_). The resulting colloidal mixture was then filtered (0.22 μm; Millipore syringe filter) to remove drug crystals and yield a non-particulate formulation.

The lomustine content of the formulations was determined by HPLC analysis of the filtrate.

### Animals

#### Ethics Statement

All animals were housed at the UCL School of Pharmacy’s Biological Services Unit (BSU) and were acclimatized in the BSU for 5–7 days before studies commenced. All animal studies were conducted in accordance with the policies and regulations of the Home Office as stipulated in the Animals and Scientific Acts 1986 UK, for the handling and care of laboratory animals used in scientific research, the recommendations of the BSU and with the approval of the ethics committee.

#### Pharmacokinetics

The MET lomustine formulation (1.04 mg ml^−1^) was intravenously administered to healthy male CD 1 mice (22–28 g) *via* the tail vein at a dose 13 mg kg^−1^ and in a dose volume of 289–357 μl. Control animals were administered an ethanolic lomustine formulation (0.37 mg ml^−1^) at a dose of 6.5 mg kg^−1^ and in a dose volume of 370–490 μl. Animals were killed at various time points and the blood, brain, liver and bone were sampled and the solid tissues stored at −80°C until analyses could be performed on them. Plasma was obtained by centrifugation of the blood samples (4000g, Hermle Z 323K centrifuge, HERMLE Labortechnik GmbH Siemensstr. 25 D-78564 Wehingen, Germany) at 4°C for 10 minutes and the plasma stored at −20°C until analyses could be performed.

#### Tissue Analysis

To thawed plasma samples (0.2 ml) was added chilled homogenising buffer [trizma HCl (50 mM), ethylene diamine tetraacetic acid [(EDTA); (0.1 mM), pH = 2, 0.2 ml], and carmustine (20 μg ml^−1^; 0.1 ml) as internal standard. This mixture was extracted with ethyl acetate (3 X 5 ml) and centrifuged (1000g) for 10 minutes at 4 ^o^C. The combined organic layers were evaporated to dryness under a stream of nitrogen.

For analysis of brain, liver and bone samples, each tissue was weighed and homogenised in homogenising buffer (1 ml) and the homogeniser rinsings (2 X 1 ml) added to the homogenate. Carmustine (20 μg ml^−1^; 0.1 ml) was added to the aqueous layer as an internal standard and the homogenate extracted with ethyl acetate (3 X 10 ml). The mixture was centrifuged (1000g) for 10 minutes at 4 ^o^C and the combined organic layers evaporated to dryness under a stream of nitrogen. The residues were subsequently reconstituted in mobile phase [acetonitrile (MeCN; HPLC grade): 0.02% ^v^/_v_ trifluoroacetic acid (TFA) in water (H_2_O): - 50: 50, 0.2 ml]. The reconstituted extracts were then subjected to HPLC analysis.

Analysis for lomustine content in the tissue samples was carried out using a gradient elution method with an initial condition of 15% MeCN in TFA (0.02% ^v^/_v_> in water) and proceeding to 65% MeCN in TFA (0.02%^v^/_v_ in water) over 10 minutes and at a flow rate of 2 mL min^−1^. The extracted samples were injected (20 μl) over an onyx monolithic C18 (5 μm, 100 x 4.6 mm) Phenomenex® column set at 40°C. Lomustine content was detected using a UV detector set at 230 nm. Calibration plot was done for plasma, brain, bone marrow and liver from which the actual concentration of lomustine in the samples was obtained. All results were expressed as mean ± standard deviation.

The pharmacokinetic parameters for the MET nanoparticle lomustine formulation was obtained using the non-compartmental method of the WinNonlin® software, version 4.1 (Pharsight Corporation, California 94040, USA).

### ***Ex-Vivo*** Coherent Anti-Stokes Raman Spectroscopy (CARS) Imaging

Deuterated N-palmitoyl-N-monomethyl-N-N-dimethyl-N,N,N-trimethyl-6-O-glycol chitosan (MET) polymer was synthesised as previously described (19) and deuterated MET particles prepared as previously described (19). Male CD-1 mice (25–30 g) were intravenously dosed with deuterated MET particles (10.4 mg ml^−1^) at a dose of 75 mg kg^−1^ and in a dose volume of 200 μl. Animals were killed 1 hour after dosing. The subsequently harvested brains were stored in neutral buffered formalin (10% ^v^/_v_). Fixed brains were cut into 0.5 mm thickness coronal slices with razor blades using a brain matrix (Zivic instruments, Pittsburgh, PA, USA). Brain slices were placed between two glass coverslips and sealed against dehydration prior to imaging using CARS microscopy, as previously described (20).

### Bone Marrow Toxicity

Male CD-1 mice were randomly assigned to treatment groups and intravenously administered (*via* the tail vein) either MET lomustine (2.02 mg ml^−1^) at a dose of 13 mg kg^−1^ in a dose volume of ≈ 200 μl or ethanolic lomustine (0.16 mg ml^−1^) at a dose of 1.2 mg kg^−1^ in a dose volume of ≈ 200 μl daily for 10 consecutive days.

Subsequently, mice were killed on 1, 7, 14, 21 or 30 days after completion of the dosing and blood and femoral marrow cell counts were assessed to determine the effect of the treatments on the bone marrow.

Blood samples were obtained by cardiac puncture and transferred into EDTA coated tubes (BD Microtainer® tube with Dipotassium EDTA; Becton, Dickson and Company, New Jersey, USA). Full blood counts were carried out using an automatic haemocytometer (Sysmex Automated Haematology Analyzer KX- 21, Sysmex Corporation, Chuo-ku, Kobe 651-0073, Japan) to determine the levels of the various blood components (white blood cells, red blood cells and platelets). Femoral cells were obtained by flushing out the bone marrow with 1 ml PBS and the cell counts were determined by flow cytometry (MACSQuant Analyzer, Miltenyi Biotec, GmbH, Germany).

### Macrophage Nanoparticle Uptake

#### Nile Red Loaded MET Nanoparticles

MET Nile Red formulations for flow cytometry studies were prepared by probe sonicating (QSonica sonicator, Connecticut, USA) Nile Red (50 μg ml^−1^) and the MET polymer (1 mg ml^−1^) in dextrose solution (5% ^w^/_v_) on ice for 30 minutes, with the instrument set at 25% of its maximum output. This was followed by centrifugation (1000g) for 30 minutes at 4 ^o^C to separate free Nile Red from encapsulated Nile Red. The supernatant was then carefully collected immediately after centrifugation. MET Nile Red formulations for confocal laser scanning microscopy (CLSM) experiments were prepared by adding a solution of Nile Red (100 μg ml^−1^, 100 μL) in ethanol to a dispersion of the MET polymer (1 mg ml^−1^) in dextrose solution (5% ^w^/_v_), to a final volume of 10 ml and probe sonicated on ice for 30 minutes, with the instrument set to 25% of its maximum output. This was followed by centrifugation (1000 g) for 30 minutes at 4^o^C to separate free Nile Red from encapsulated Nile Red.

#### Nile Red Loaded Liposomes

Nile Red (50 μg ml^−1^), egg phosphatidyl choline (3 mg ml^−1^) and cholesterol (1.4 mg ml^−1^) were dissolved in chloroform (10 ml) and the resulting solution evaporated to dryness at 40°C using a rotary evaporator. The thin lipid film obtained was then hydrated with 5% ^w^/_v_ dextrose solution (5 ml) by shaking for 30 minutes at room temperature to yield a homogenous dispersion of egg phosphatidyl choline liposomes. This dispersion was subsequently probe sonicated on ice for 15 minutes, as described above. The liposome formulation was then centrifuged (1000g) for 30 minutes at 4 ^o^C to eliminate free unencapsulated Nile Red and the supernatant was carefully collected immediately after centrifugation. These liposomes were used for the flow cytometry studies.

Liposomal Nile Red formulations for CLSM imaging were prepared by adding a Nile Red solution (100 μg ml^−1^, 100 μL) in chloroform to egg phosphatidyl choline (3 mg ml^−1^) and cholesterol (1.4 mg ml^−1^) dissolved in chloroform (10 ml) and following the methodology outlined above.

For both the Liposome and MET Nile Red formulations used for CLSM, the concentration of Nile Red [determined by fluorimetry, λexc = 488 nm and λem = 655 nm, (LS – 50 B, Perkin Elmer Inc., USA Spectrofluorimeter, with FL WinLab (Perkin Elmer Inc, USA) software] was adjusted so that both formulations had the same Nile Red concentrations (0.2 μg ml^−1^) prior to application to the cells.

#### Cell Uptake Experiments

J774A.1 cells were cultured in Dulbecco’s Modified Eagle’s Medium [(DMEM; ATCC® 30-2002™) modified to contain L-glutamine (4 mM), glucose (4.5 g L^−1^), sodium pyruvate (1 mM), and 1500 mg L^−1^ sodium bicarbonate] supplemented with 10% fetal bovine serum. The cells were maintained in culture for at least 14 days before they were used for uptake experiments. For flow cytometry and confocal imaging, cells were handled according to methods described by Fernando *et al*. 2010 (21) and Kim *et al*. 2012 (22) with some modifications, as outlined below.

Cells were seeded in 6 well plates at a density of 300,000 cells per well and incubated for 72 hours. Cells were then treated with either MET Nile Red (0.3 μg ml^−1^) or the liposome Nile Red (0.4 μg ml^−1^) formulations with some wells left untreated as control. Cells were treated for predetermined time periods of 5, 10, 30 minutes, 1 hour, 2 hours, or 4 hours. Cells were then washed 3 times with cold Dulbecco’s phosphate buffered solution (DPBS, [calcium chloride anhydrous (CaCl_2_; 0.9 mM), magnesium chloride (MgCl_2_.6H_2_O; 0.5 mM), potassium chloride (KCl; 2.7 mM, potassium phosphate monobasic (KH_2_PO_4_; 1.5 mM), sodium chloride (NaCl 137.9 mM), sodium phosphate dibasic (Na_2_HPO_4_.7H_2_O; 8.1 mM), pH 7.0–7.2] (2 ml / well) and then incubated in cold PBS – EDTA (PBS- EDTA, [KH_2_PO4 (1.9 mM), NaCl (138.9 mM), Na_2_HPO4.2H_2_O (6.6 mM), Titriplex III (EDTA Na_2_; 1.4 mM), lithium chloride (LiCl; 10.1 mM); pH 7.5)] (1 ml / well) for 1–2 minutes. Cells were then harvested by gently scraping and pipetting the cell suspension which was then centrifuged and suspended in cold Ringers solution [Sodium chloride (38.5 mM), Potassium chloride (1.4 nM), Calcium chloride hexahydrate (0.5 mM), Sodium bicarbonate (0.6 mM); pH 7] and the nanoparticle uptake was quantified using the flow cytometer (MACSQuant Analyzer, Miltenyi Biotec, Germany) with uptake quantified with reference to the Nile Red fluorescence. A total of 20,000 cells were measured in each sample within the Nile Red positive channel. The experiment was carried out in quadruplet for each formulation and the untreated control cells.

For confocal microscopy imaging, cells were seeded in glass bottom 35 mm tissue culture dishes (MatTek, Corporation, Ashland, MA, USA) at a density of 75,000 cells per dish and incubated for 48 hours. Cell uptake of the formulations was then monitored in a time-lapse experiment set up on a Zeiss LSM 710 laser scanning microscopy imaging unit (LASOS Lasertechnik GmbH, Carl Zeiss, Franz-Loewen-Straße 2, 07745 Jena, Germany). Images were captured 3 minutes after treatment with the formulation and subsequently after every 5 minutes. The images were analysed using Zen 2009 software (Carl Zeiss Microscopy GmbH Carl Zeiss Promenade 10, 07745 Jena, Germany).

### Brain Tumour Studies

#### Brain Tumour Model and Magnetic Resonance Imaging

Tumour bearing mice brains were embedded in a mixture (50: 50) of agarose gel (1% ^w^/_v_) and formaldehyde solution (4% ^w^/_v_) prior to imaging, to fix the tissue and minimize shifting of the samples during measurement. Images were collected using a 1.5 T Philips Intera Gyroscan magnet (Philips Healthcare, 5680 DA Best, The Netherlands). A high resolution T2-weighted Turbo Spin-Echo (TSE) sequence was used (Repetition time (TR) = 3000 ms, Echo time (TE) = 110 ms, flip angle = 90 degrees, number of slices = 54, voxel size = 0.14 X 0.14 X 0.14 mm^3^, 10 averages). High-resolution images were achieved by utilizing a microscopy coil, diameter 23 mm.

Image parameters were established in a preliminary experiment, so as to optimize tumour edge detection. OSIRIX software (Pixmeo SARL, Switzerland) was utilized to analyse the MRI images and to measure the tumour volume, after a manual determination of the edges in a slice-by-slice process.

#### Brain Tumour Treatment

Human glioblastoma (U-87 MG) cells were cultured in Minimum Essential Medium (MEM) supplemented with foetal bovine serum (FBS, 10% ^w^/_v_); sodium pyruvate (1 mM; 5 ml) and L-Glutamine (1% ^w^/_v_; 5 ml). Cells were maintained in culture for up to 14 days before they were used for tumour implantation.

Intracranial tumour models were established by orthotopic implantation of the glioblastoma cells (100,000 cells) in the left striatum (+0.5 mm anterior, – 2 mm lateral and to a depth of 3mm to the bregma, determined by a BENCHmark™ digital stereotaxic control panel), of stereotactically fixed anaesthetised (inhaled isoflurane) female CD-1 nude mice (20–30g in weight). Once tumours were established (7 days after implantation) mice were randomly assigned to treatment groups and injected daily (tail vein) with the lomustine formulations. Animals were either dosed with MET lomustine (2.6 mg ml^−1^) at a dose of 13 mg kg^−1^ and a dose volume of ≈ 200 μL or were dosed with ethanolic lomustine (0.18 mg ml^−1^) at a dose of 1.2 mg kg^−1^ and a dose volume of ≈ 200 μL. Animals were dosed on 10 consecutive days and the dose of the ethanolic lomustine formulation was the maximum dose volume that could be administered with repeat dosing. Animals were weighed daily and animals were killed once body weight had declined by 15% compared to the start of treatment. Examination of control animals once body weight had reached this threshold value revealed tumours which were 83.4 – 98.1 mm^3^ in volume, with a mean volume of 91.8 ± 7.57 mm^3^

### Statistics

Data were subjected to a one-way analysis of variance (ANOVA) with Tukey post hoc test using SPSS, version 17.0 software (SPSS Inc., Chicago, USA) for the pharmacokinetics experiment and Minitab® 16 software (Minitab, Inc., Pennsylvania, USA) for the brain tumour and bone marrow toxicity experiments. The treatment groups were compared two groups at a time for each time point and the significance level was set at 0.05.

## Results

### Synthesis of the MET Polymer

Nanomerics’ MET is based on the self-assembling polymer N-palmitoyl-N-monomethyl-N,N-dimethyl-N,N,N-trimethyl-6-O-glycol chitosan, which assembles into nanoparticles in aqueous media (17,18). Various batches of the MET polymer were synthesised and characterised (Table I).

### Preparation of MET Lomustine Nanoparticles

**Fig. 1:**
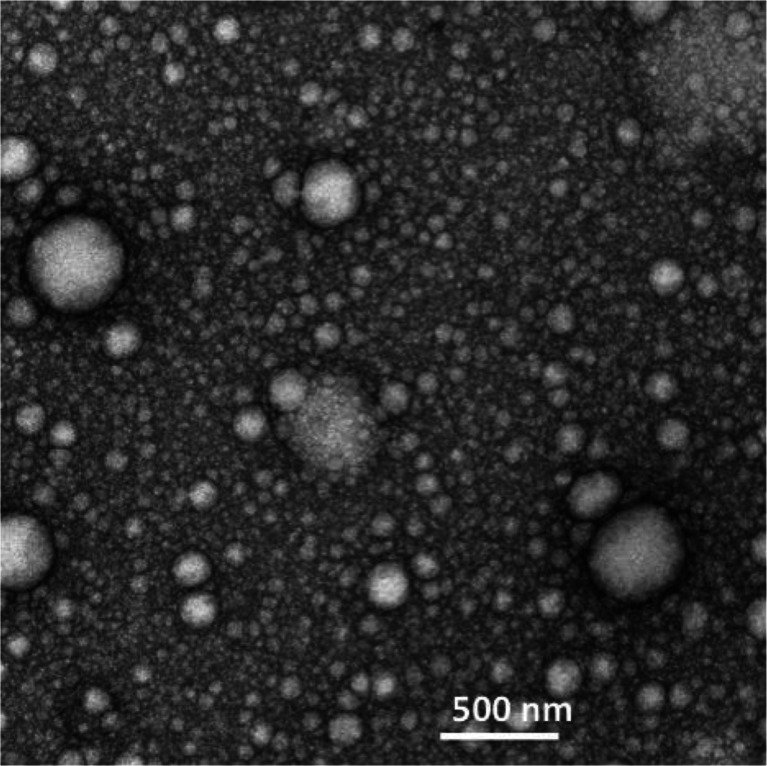
TEM image with negative staining of a lomustine formulation containing lomustine (2 mg ml^−1^), soya bean oil (10 mg ml^−1^), polysorbate 80 (5 mg ml^−1^), MET polymer batch GCPQOO28112009 [(mol % palmitoylation = 22 and mol % quaternisation = 8%; 20 mg ml^−1^)] in dextrose (5% *w*/*v*). Formulations were prepared by reconstituting a freeze dried sample containing 0.77 mg ml^−1^ lomustine to half its original volume.

The nanoparticle formulations presented as translucent liquids with a z-average mean

particle size of 336 ± 0.44 nm and a polydispersity of 0.5. The formulations were relatively polydisperse. Nanoparticles were spherical, presumably consisting of lomustine filled oil droplets in an oil in water formulation (Fig. 1) as well as MET polymer and polysorbate 80 micelles. The oil droplets were stabilised by the MET polymer and polysorbate 80. The oil droplets/ micelles (it was not possibly to conclusively distinguish both particle types using electron microscopy) varied in size from as little as 50 nm to up to 600 nm in size. Although the majority of the oil droplets and micelles were below 100 nm in size, the PCS method of particle size analysis is heavily weighted towards the larger sized particles. This is the first report of Nanomerics’ MET acting as a stabiliser of emulsion formulations and forming nanoemulsions.

**Fig. 2:**
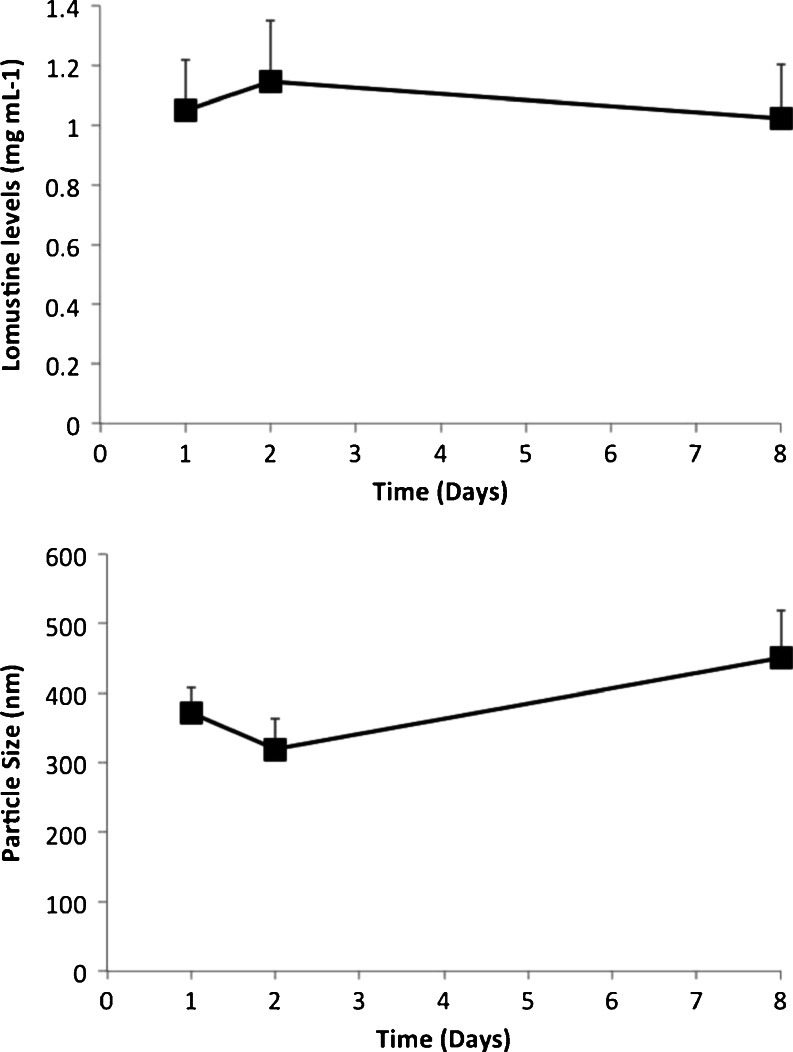
The stability of MET-lomustine nanoparticle formulations when stored as the freeze dried cake at room temperature. Formulations were reconstituted in water prior to analysis and no crystals were observed after reconstitution.

The nanoemulsions were stable for up to 8 days when stored dry at room temperature and could be reconstituted into nanoemulsions (Fig. 2), with no sign of drug crystal formation (data not shown).

### *Ex-Vivo* CARS Imaging

**Fig. 3:**
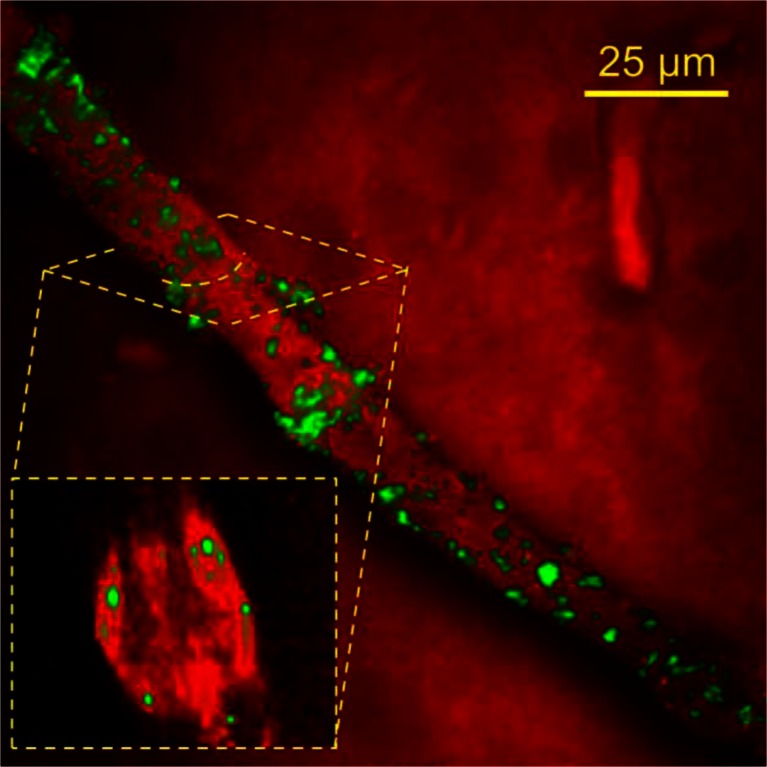
Epi-detected CARS microscopy image illustrating the distribution of deuterated MET nanoparticles within a mouse brain blood vessel, from a sample harvested 1 hour after intravenous injection of deuterated MET nanoparticles (75 mg kg^−1^). The pump and Stokes wavelengths were tuned to probe the C-D resonance at 2100 cm^−1^ (*green contrast*) and the C-H resonance at 2845 cm^−1^ (*red contrast*).

CARS microscopy imaging detects clusters of polymer molecules. The intensity of the CARS signal scales quadratically with the concentration of bonds being probed within the focal volume (23). Therefore, clusters of polymers in a single nanoparticle will generate significantly higher signal than individual polymer molecules. The signal strength of the carbon-deuterium CARS signal in Fig. 3 was several orders of magnitude greater than the non-resonant background, and therefore was indicative of nanoparticles, rather than individual polymer chains (24).

The deuterium labelled MET nanoparticles were visualised in *ex-vivo* brain samples after intravenous injection and were found to adhere to the brain endothelium, *i.e.* to the luminal side of the blood brain barrier (BBB). The MET polymer CARS signal was not seen in the brain parenchyma and as previously reported particles appear to adhere to the BBB (20) and are not delivered to the brain (16). However, MET nanoparticles are delivered to the BBB, adhere to the brain endothelial cells (Fig. 3), from where they presumably release loaded drug for transport across the BBB.

### Pharmacokinetics

**Fig. 4:**
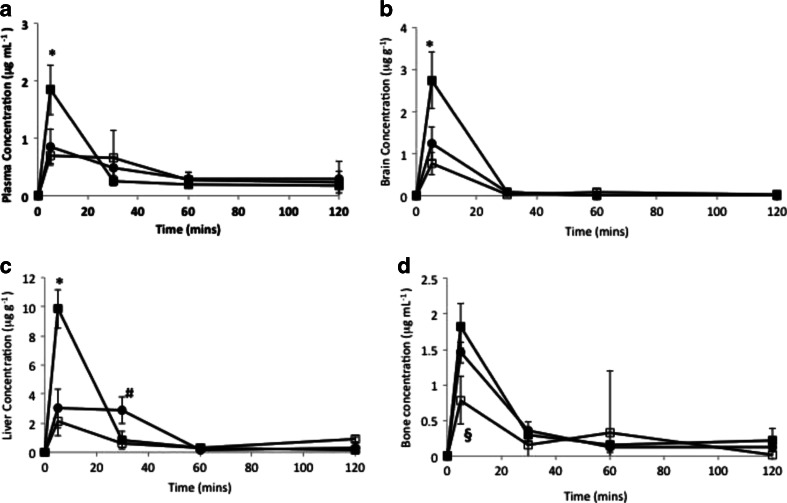
The biodistribution of MET - lomustine nanomedicine formulations: a = plasma, b = brain, c = liver, d = bone (including bone marrow), ■ = MET – lomustine (13 mg kg^−1^), □ MET - lomustine (6.5 mg kg^−1^), ● = ethanolic lomustine (6.5 mg kg^−1^). The MET formulation improves the delivery of lomustine to the brain by 2 fold by enabling a higher dose to be administered. Additionally, when compared to an ethanolic formulation of the drug at a dose of 6.5 mg kg^−1^, the MET nanoparticles reduces the exposure (AUC_0 – 4h_) of the bone marrow and liver to lomustine by 25 and 38% respectively, * = significant difference between the high dose MET formulation (13 mg kg^−1^) and all other formulations (*p* < 0.05), # = significant difference between ethanolic formulation and both MET formulations (*p* < 0.05), § = significant difference between low dose MET formulation (6.5 mg kg^−1^) and all other formulations (*p* < 0.05).

Following intravenous administration, plasma levels of lomustine were relatively low with less than 1% of administered dose detected in the plasma after 5 minutes (Fig. 4). Lomustine is rapidly distributed to tissues and metabolised and is not normally detected in the plasma (25).

The MET formulation improves the delivery of lomustine to the brain by 2 fold by enabling a higher dose to be administered. Brain levels were similar to plasma levels for all formulations, evidence of good across BBB transport for the drug (Fig. 4, Tables II, III and IV). Additionally, when compared to an ethanolic formulation of the drug at a dose of 6.5 mg kg^−1^, the MET nanoparticles reduce the exposure (AUC_0 – 4h_) of the bone (which includes the bone marrow) and liver to lomustine by 25 and 38% respectively, whereas brain exposure (AUC_0 – 4h_) for both formulations is similar (Fig. 4 and Table II). The Cmax of the bone is also higher for the 6.5 mg kg^−1^ ethanolic formulation when compared to the 6.5 mg kg^−1^ MET formulation (Fig. 4d, Table IV). A doubling of the lomustine dose by administering lomustine in the form of the MET formulations (6.5mg kg^−1^ ethanolic lomustine injection vs 13 mg kg^−1^ MET - lomustine) results in virtually no change in the plasma AUC_0-120_, presumably because

**Table II:** Lomustine Tissue AUC_0-120_ Following Intravenous Dosing

Formulation and dose	Dose	Brain AUC_0-120min_ (μg g^−1^ min)	Bone AUC_0-120min_ (μg g^−1^ min)	Liver AUC_0-120min_ (μg g^−1^ min)	$\frac{BrainAU{C}_{o-120 \min }}{BoneAU{C}_{o-120 \min }}$	$\frac{BrainAU{C}_{o-120 \min }}{LiverAU{C}_{o-120 \min }}$
MET - Lomustine	13 mg kg^−1^	44.06	49.03	187.18	0.899	0.235
Ethanolic lomustine	6.5 mg kg^−1^	21.54	40.89	141.10	0.527	0.152
MET - Lomustine	6.5 mg kg^−1^	17.28	30.79	87.64	0.561	0.197

**Table III:** Lomustine Plasma AUC_0-120_ Following Intravenous Dosing

Formulation	MET - Lomustine	Ethanolic lomustine	MET - Lomustine
Dose	13 mg kg^−1^	6.5 mg kg^−1^	6.5 mg kg^−1^
Plasma AUC_0-120_ (μg ml^−1^ min)	48.35	48.21	48.33
$\frac{BrainAU{C}_{o-120 \min }}{PlasmaAU{C}_{o-120 \min }}$	0.91	0.45	0.36
$\frac{LiverAU{C}_{o-120 \min }}{PlasmaAU{C}_{o-120 \min }}$	3.87	2.93	1.81
$\frac{BoneAU{C}_{o-120 \min }}{PlasmaAU{C}_{o-120 \min }}$	1.01	0.848	0.637

**Table IV:** Lomustine Cmax Values

Formulation	Dose (mg kg^−1^)	Plasma (μg ml^−1^) mean ± s.d	Brain (μg g^−1^) mean ± s.d	Liver (μg g^−1^) mean ± s.d	Bone (μg g^−1^) mean ± s.d
MET lomustine	13	1.84 ± 0.43*	2.74 ± 0.67*	9.85 ± 1.32*	1.82 ± 0.31
Ethanolic lomustine	6.5	0.84 ± 0.32	1.24 ± 0.40	3.00 ± 1.33	1.46 ± 0.15
MET lomustine	6.5	0.70 ± 0.15	0.76 ± 0.27	2.12 ± 0.98	0.78 ± 0.34*

* = significant differences between formulation and all other formulations (*p* < 0.05)

lomustine is extensively distributed and metabolised in the plasma (25), but does lead to a proportional increase in brain AUC_0-120_ values (2.2 fold) and a limited increase in both bone (1.2) and liver (1.3) AUC_0-120_ values (Tables II and III).

At the highest dose, MET lomustine delivers 0.33% (0.77% per gram of brain) of the dose to the brain. It is clear that the MET formulation produces higher brain levels, while sparing the bone marrow and liver and this altered distribution is not underpinned by an increase in plasma exposure (as is seen with other pharmaceutical particulates such as liposomes (26,27)) but is due to differential tissue uptake mechanisms.

Animals administered 6.5 mg kg^−1^ lomustine either as the MET or ethanolic formulation showed no difference in the drug’s plasma and brain Cmax, whereas the ethanolic formulation produced a significantly higher Cmax in the bone (including the bone marrow), when compared to the MET formulation (Fig. 4, Table IV), we thus conclude that the MET particles avoid the bone and bone marrow. Furthermore, with the 6.5 mg kg^−1^ dose, the MET particles are cleared faster from the liver when compared to the drug injected as an ethanolic solution (Fig. 4c) and we thus conclude that the MET particles are not retained by the liver. Lomustine chemotherapy is associated with dose limiting myelosuppression (15,28–30) and hepatotoxicity in dogs (31). Myelosuppression and hepatotoxicity are thus dose limiting adverse effects for the drug and any formulation that minimises bone marrow and liver exposure should have a positive impact on treatment.

### Bone Marrow Toxicity

**Fig. 5:**
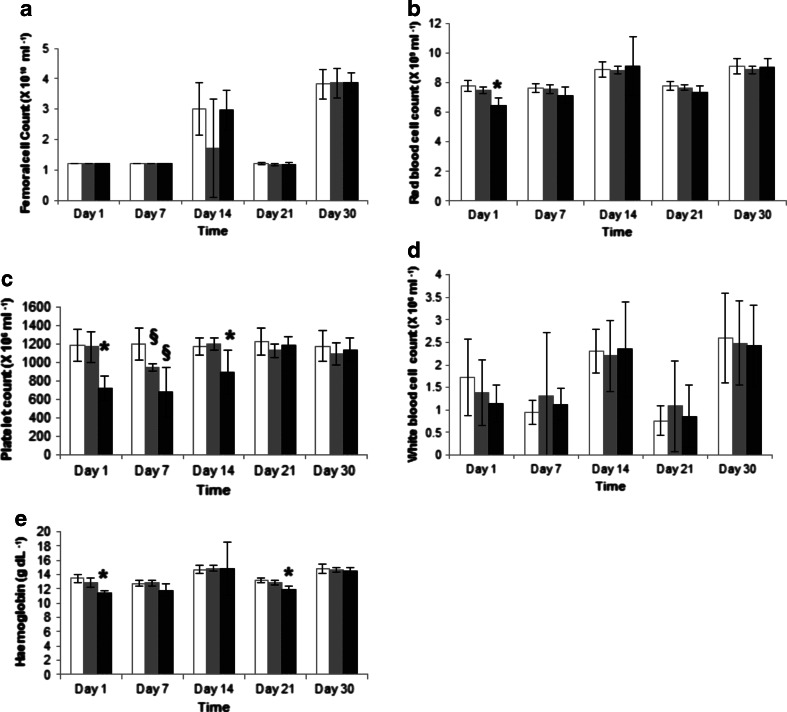
Mice blood cell counts and haemoglobin assessments following 10 daily doses of lomustine formulations: *white bar* = control animals, *grey bar* = ethanolic lomustine formulation (1.2 mg kg^−1^), *black bars* = MET lomustine (13 mg kg^−1^), * = statistically significantly different from all formulations, § = statistically significantly different from control animals (untreated).

Following the observation that the MET formulation delivered proportionately less lomustine to the bone and bone marrow, an assessment of bone marrow toxicity was carried out (Fig. 5) after chronic dosing of either MET lomustine (13 mg kg^−1^) or an ethanolic formulation of lomustine at the highest dose possible with multiple dosing (1.2 mg kg^−1^).

Full femoral cell counts (Fig. 5a) and white blood cell counts (Fig. 5d) remained unchanged for both formulations.

There were minor changes in red cell count (83.0 ± 8.0% of control values) and haemoglobin levels (84.5 ± 3.5% of control values) with the high dose (13 mg kg^−1^) MET formulation (Fig. 5b and e) on Day 1 with recovery after 7 days for the red cell count and recovery after 30 days for the haemoglobin levels. The changes in haemoglobin levels are unlikely to be clinically significant as the range of acceptable values for haemoglobin [male 14–18 g/dL; female 12–16 g/dL (32) spans at least a ± 20% range.

The main myelosuppressive changes were detected in the platelet count, with platelet levels falling for both the low dose ethanolic (1.2 mg kg^−1^) and high dose MET (13 mg kg^−1^) formulation treated groups (Fig. 5c). The nadir values for both groups were recorded on Day 7 with platelet nadir values of 56 and 79% of the control values recorded for the high dose MET (13 mg kg^−1^) and low dose (1.2 mg kg^−1^) ethanolic formulations respectively. For the ethanolic formulation treated animals, recovery was observed after 14 days while the higher dose MET nanoparticle treated group showed recovery after 21 days. In essence the administration of ten times the ethanolic dose as a MET formulation resulted in very little additional haematological toxicity when compared to the low dose ethanolic formulation.

### Macrophage Uptake

Macrophages are a heterogeneous group of cell types, which are resident in a number of tissues, including the bone marrow and the liver; with one of their functions being the removal of particulate cellular debris (33). In order to understand the mechanisms underpinning the low liver, bone and bone marrow drug levels, we hypothesised that the main mechanism could lie with a reduced uptake of the MET nanoparticles by the macrophages in the liver and bone marrow. Previously we have shown that on intravenous injection, liver deposition is comparatively low for MET nanoparticles, with only 4% of the intravenous dose found in the liver 10 minutes after dosing (16), compared to peak levels of 67% of the intravenous dose of liposomes found in the liver 80 minutes after dosing (34). We hypothesised that the low liver uptake of MET nanoparticles could be due to the low uptake by liver macrophages and it is conceivable that the bone levels may also be reduced due to low bone marrow macrophage uptake.

**Fig. 6:**
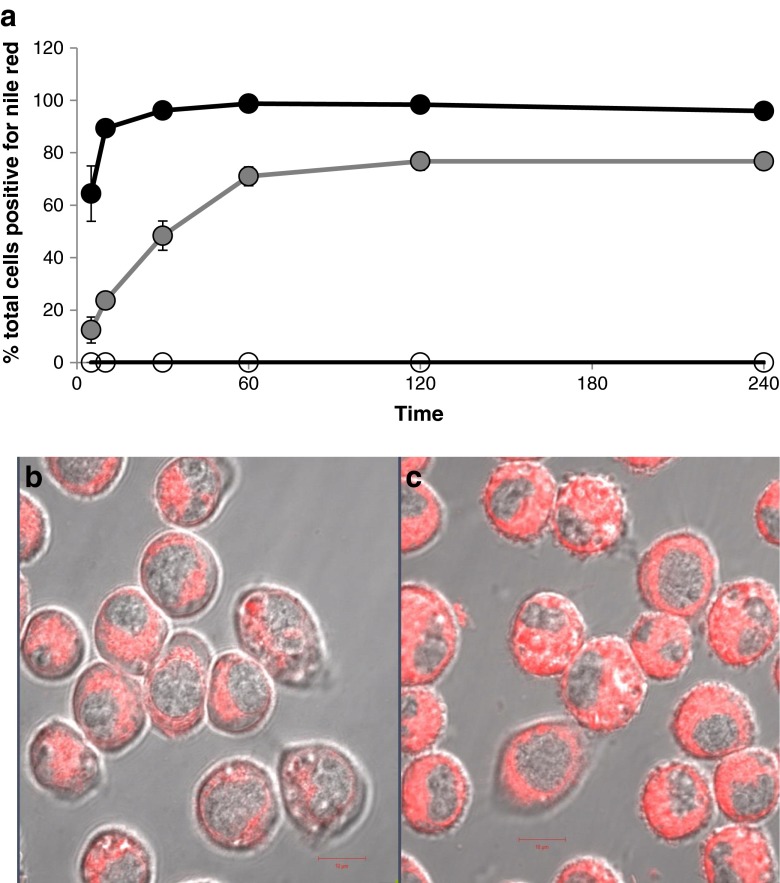
(**a**) The uptake of Nile red loaded particles in the J774A.1 cell line (a macrophage cell line). Cells were treated with Nile red loaded MET nanoparticles or Nile red loaded liposomes at a Nile red concentration of 0.3 μg ml^−1^ and 0.4 μg ml^−1^ respectively. The cells were washed to remove excess formulation prior to analysis. (**b**): Confocal laser scanning microscopy image of J774A.1 cells following incubation with MET Nile Red particles (0.2 μg ml^−1^) for 18 minutes. (**c**) Confocal laser scanning microscopy image of J774A.1 cells following incubation with liposomal Nile Red (0.2 μg ml^-1)^ for 18 minutes. *Scale bar* = 10 μm.

*In vitro* within the J774A.1 cell line, the uptake of liposomal Nile Red is rapid with 89% of cells positive for Nile Red within 10 minutes of incubation (Fig. 6a). While MET nanoparticles are also taken up by macrophages, uptake is significantly slower with 71% of cells positive for MET Nile Red one hour after incubation. Uptake as opposed to a simple cell surface association was verified by confocal laser scanning microscopy, which shows the Nile Red signal at the level of the nucleus (Fig. 6b and c).

The rate of macrophage uptake is thus slower for the MET nanoparticles when compared to the uptake by liposomes and it is thus possible that the reduced liver and bone and bone marrow drug levels and consequent relatively mild effect on the bone marrow of the dosage form could be the result of reduced macrophage uptake. The macrophage uptake data thus provides further proof that the MET nanoparticles appear not to be taken up by the reticuloendothelial system to an appreciable extent.

### Brain Tumour Model

**Fig. 7:**
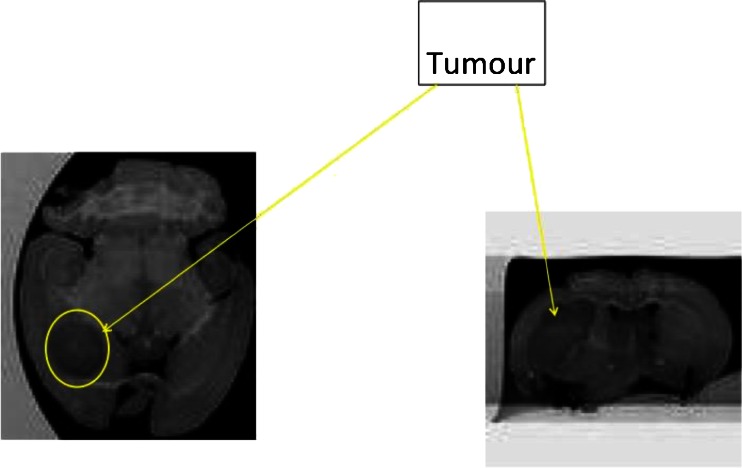
Magnetic resonance T_2_ weighted images of an established intracranial tumour.

U-87 MG intracranial tumours were successfully established and imaged by MRI (Fig. 7).

### Brain Tumour Treatment

**Fig. 8:**
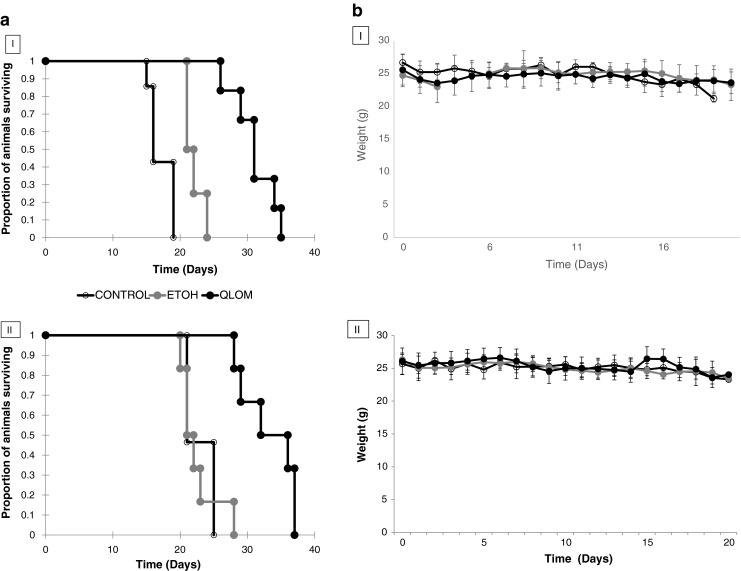
(**a)** I (*top*- first study) and II (*bottom*- second study): Survival plots of female CD-1 mice bearing an intracranial U-87 MG tumour, following treatment with: **a**) MET lomustine (13 mg kg^−1^ per day 2.6 mg ml^−1^, dose volume = ≈ 200 μL, *black filled symbols*), **b**) ethanolic lomustine (1.2 mg kg^−1^ per day; 0.18 mg ml^−1^, dose volume = ≈ 200 μL, *grey filled symbols*) or **c**) untreated (*unfilled symbols*). Mice were killed once body weight had declined by 15% compared to the start of treatment. Mice were administered 10 doses with treatment commencing on Day 7 and treatment concluded on Day 16. (**b**) (I and II): Body weight monitoring of female CD-1 mice bearing intracranial U-87 MG tumours following treatment with lomustine formulations: *filled black symbols* = MET lomustine (13 mg kg^−1^ per day, 2.6 mg mL^−1^, dose volume = ≈ 200 μL, *filled grey symbols* = ethanolic lomustine (1.2 mg kg^−1^ per day, 0.18 mg mL^−1^, dose volume = ≈ 200 μL), *unfilled symbols* = untreated control animals. Body weights were not significantly different during the treatment phase of the experiment.

Animals with established tumours (7 days after implantation of the U-87 MG cells) were treated for 10 consecutive days, with treatment commencing on Day 7 and concluding on Day 16. The mean survival time for animals that received the MET lomustine (13 mg kg^−1^ per day) formulation was 33.17 days, while untreated control animals had a mean survival time of 21.33 days (Fig. 8a II). A comparable mean survival time (31 days) was obtained for the MET lomustine formulation treated animals in a previous (first) study. The untreated control animals in the first study had a mean survival time of 17.14 days (Fig. 8a I). Animals that received the ethanolic lomustine (1.2 mg kg^−1^) formulation of the drug had similar mean survival times of 22 and 22.5 days in the first and second studies respectively (Fig. 8a I and II). The dose of lomustine (1.2 mg kg^−1^) administered in the ethanolic formulation was limited by the poor aqueous solubility of lomustine and was the dose achievable with multiple dosing when lomustine was formulated in 10% ^v^/_v_ ethanol containing polysorbate 80 (5 mg ml^−1^).

During treatment the animals’ body weight was monitored and there was no significant difference between the body weights when all groups were compared (untreated, ethanolic and MET treated animals – Fig. 8b). This indicates that the high dose formulation did not cause gross toxicities and the MET formulation thus enabled the administration of a higher dose, with the lack of uptake by the bone marrow and liver likely to limit the toxic effects (15,28–30) that are normally observed with lomustine therapies.

**Fig. 9:**
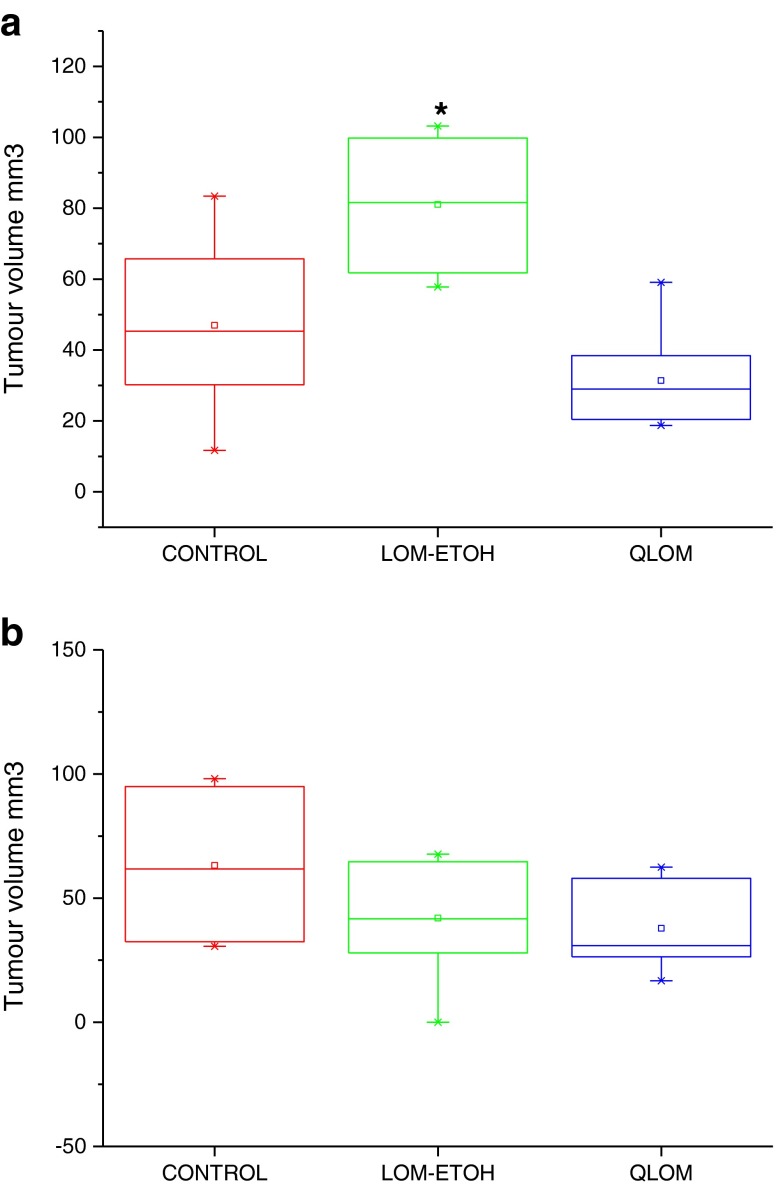
(**a)** (*top*- first study) and (**b**) (*bottom*- second study): Tumour volumes for female CD-1 mice bearing intracranial U-87 MG tumours following treatment with lomustine formulations. QLOM = MET lomustine (13 mg kg^−1^ per day, 2.6 mg mL^−1^, dose volume = ≈ 200 μL, LOM-ETOH = ethanolic lomustine (1.2 mg kg^−1^ per day, 0.18 mg mL^−1^, dose volume = ≈ 200 μL), CONTROL = untreated control animals. * = Significantly different from MET lomustine (13 mg kg^−1^) formulation treated animals.

MRI analysis showed that tumour volumes for the MET lomustine formulation treated animals in the first study were significantly (p < 0.05) lower than those for the ethanolic formulation treated animals (Fig. 9a). However, there was an outlier with a tumour volume considerably larger than the others within the group of animals that had received the ethanolic formulation. This necessitated the conduct of the second experiment to confirm the findings from the first study. Analysis of the tumour volumes in the second experiment confirmed that tumour volumes for animals that received the MET lomustine formulation were generally lower than those of animals that received the ethanolic formulation and untreated control animals (Fig. 9b). However, the differences were not statistically significant (p > 0.05).

## Discussion

The MET formulation presented as a nanoemulsion in which the oil droplets are presumably coated with the MET polymer and polysorbate 80 (Fig. 1). The size distribution is heterogeneous and comprises micelles as well as oil droplets. The distribution of the two amphiphiles—the MET polymer and polysorbate 80 between the micelles and the oil droplets is unknown. This formulation enables a higher dose to be administered as a bolus and this is in turn associated with a longer survival time (Fig. 8a), when compared to the administration of the lomustine ethanolic—polysorbate 80 formulation at the maximum bolus dose possible. However, it is the relatively reduced delivery of drug to the liver and bone (Fig. 4, Tables II and III) and the resultant minimal effect of the high dose formulation on myelosuppression (Fig. 5) that is the most interesting feature of the MET formulation. Lomustine chemotherapy is associated with dose limiting myelosuppression (15,28–30) and autologous stem cell rescue (15). Hepatotoxicity in dogs is also a feature of lomustine chemotherapy (31). Myelosuppression and hepatotoxicity are thus dose limiting adverse effects for lomustine and any formulation that minimises bone marrow and liver exposure would have a positive impact on treatment and allow patients to take fewer treatment breaks, as the tumour would presumably continue to grow during such treatment breaks allowing the supporting microenvironment to recover rapidly. Lomustine is normally given orally and we administered it intravenously in this study, using it as a model drug to test the bone marrow sparing potential of the intravenous MET formulation.

We have found that MET nanoparticles are taken up at a slower rate *in vitro* by mouse monocyte derived macrophage cell lines (Fig. 6) and while these are not tissue resident macrophages which have distinct functions, such as immune surveillance, removal of cell debris and iron processing (33), it is clear that these macrophages phagocytose liposomes more readily than when compared to the MET nanoparticles and we conclude that the surface chemistry of the MET particles must be responsible for their slower phagocytosis when compared to liposomes. Such a conclusion is not without precedent as the exposure of phosphatidyl serine on the surface of apoptotic cells enables these cells to be taken up by tissue resident macrophages expressing the phosphatidyl serine receptor (33).

It is possible that this reduced macrophage uptake is a key driver for the low bone and liver levels of the drug (Fig. 4). Low liver uptake of MET nanoparticles has been demonstrated in a number of studies (16,35). For example the coating of peptide nanofibres with the MET polymer resulted in a significant reduction in liver deposition (35), hence evidence is beginning to emerge in support of the hypothesis that the MET polymer coating diverts nanoparticles from the liver (Fig. 4), spleen (16) and bone marrow (Fig. 4) on intravenous injection. While it is clear that there are correlations between macrophage uptake (Fig. 6) and liver (Fig. 4) and spleen (16) deposition of MET nanoparticles, when compared to liposomes (34) (Fig. 6), we have not proven that the reduced macrophage uptake observed actually leads to reduced liver, spleen and bone marrow deposition of MET nanoparticles on intravenous injection, especially as tissue resident macrophages (which are proliferative and of embryonic origin) (33) were not used in our *in vitro* studies.

To further understand the advantages offered by the liver/ bone sparing MET formulation, which in essence targets the drug to the brain, one must consider the fact that dose intensification would allow the drug to overwhelm the enzyme repair systems that confer resistance to the therapeutic, such as the enzyme O^6^-methylguanine-DNA methyltransferase (MGMT), a DNA repair enzyme (7). Dose intensification, when tried in the clinic, although beneficial, is usually confounded by the toxicity of the regimen, most notably haematological toxicities (36). In our studies, dose intensification with the MET formulation does not confer significant additional myelosuppressive effects when compared to the low dose ethanolic formulation and yet is therapeutically beneficial in this mouse intracranial tumour model. Dose intensification with the MET system definitely warrants clinical testing.

Dose intensification with chemotherapy may be achieved either by increasing the dose or by increasing the frequency of dosing. The resulting haematological toxicities will take the form of neutropenia, thrombocytopenia or a reduction in red blood cells (36,37). Neutropenia, may lead to fatal infections and is the most frequently occurring toxic effect encountered with cytotoxic drugs (37). Due to the short life span (6–14 hours) (38) of granulocytes, they are usually the first to be negatively affected during chemotherapy (38). However, in the current study, the white blood cells were not affected by the high dose MET formulation and neither were the total bone marrow cells (Fig. 5a and d). Platelets with a life span of 9–10 days in humans (39) and 4–5 days in the mouse (40) are usually the next susceptible cell type to be affected by chemotherapy after the granulocytes (41). The results of the current study are consistent with this platelet vulnerability, as all treatment groups show a significant drop in platelet counts after 7 days, although platelets counts did revert to baseline levels after 14 and 21 days for the low dose ethanolic (1.2 mg kg^−1^) and high dose MET (13 mg kg^−1^) formulations, respectively (Fig. 5c).

Toxic effects on the red blood cells are the last to be seen due to the longer life span of the erythrocytes with a life span of 100–120 days in humans (41,42) and 30–52 days in the mouse (40) and although there were minor drops recorded in the red cell counts and haemoglobin levels in our studies, these changes in red blood cell counts and haemoglobin levels amount to no more than a 20% change and thus are unlikely to be clinically significant.

## Conclusion

Our findings show that lomustine dose intensification with the MET particle system improved the survival of intracranial tumour bearing mice and did not produce significant additional myelosuppressive effects as bone and bone marrow deposition of the drug was reduced with the MET system. Liver deposition was also reduced with the MET system and since myelosuppression and liver toxicity are features of lomustine therapy, such a dose intensification strategy warrants clinical testing.

## Abbreviations

CLSM: Confocal laser scanning microscopy
EDTA: Ethylene diamine tetraacetic acid
GBM: Glioblastoma multiforme
GCPQ: N-palmitoyl-N-monomethyl-N-N-dimethyl-N,N,N-trimethyl-6-O-glycol chitosan
HPLC: High performance liquid chromatography
MeCN: Acetonitrile
MET: Molecular envelope technology
MRI: Magnetic resonance imaging
TFA: Trifluoroacetic acid

## References

[1] Ohgaki H, Kleihues P (2005). Epidemiology and etiology of gliomas. Acta Neuropathol.

[2] Ohgaki H, Dessen P, Jourde B, Horstmann S, Nishikawa T, Di Patre PL (2004). Genetic pathways to glioblastoma: a population-based study. Cancer Res.

[3] Grossman SA, Ye X, Piantadosi S, Desideri S, Nabors LB, Rosenfeld M (2010). Survival of patients with newly diagnosed glioblastoma treated with radiation and temozolomide in research studies in the United States. Clin Cancer Res.

[4] Krex D, Klink B, Hartmann C, von Deimling A, Pietsch T, Simon M (2007). Long-term survival with glioblastoma multiforme. Brain : J Neurol.

[5] Kesari S (2011). Understanding glioblastoma tumor biology: the potential to improve current diagnosis and treatments. Semin Oncol.

[6] Omuro AM, Leite CC, Mokhtari K, Delattre JY (2006). Pitfalls in the diagnosis of brain tumours. Lancet Neurol.

[7] van den Bent MJ, Hegi ME, Stupp R (2006). Recent developments in the use of chemotherapy in brain tumours. Eur J Cancer.

[8] Khasraw M, Lassman AB (2010). Advances in the treatment of malignant gliomas. Curr Oncol Rep.

[9] Agarwal S, Manchanda P, Vogelbaum MA, Ohlfest JR, Elmquist WF (2013). Function of the blood-brain barrier and restriction of drug delivery to invasive glioma cells: findings in an orthotopic rat xenograft model of glioma. Drug Metab Dispos.

[10] Agarwal S, Sane R, Ohlfest JR, Elmquist WF (2011). The role of the breast cancer resistance protein (ABCG2) in the distribution of sorafenib to the brain. J Pharmacol Exp Ther.

[11] Chamberlain MC (2010). Temozolomide: therapeutic limitations in the treatment of adult high-grade gliomas. Expert Rev Neurother.

[12] Lonardi S, Tosoni A, Brandes AA (2005). Adjuvant chemotherapy in the treatment of high grade gliomas. Cancer Treat Rev.

[13] Gerber DE, Grossman SA, Zeltzman M, Parisi MA, Kleinberg L (2007). The impact of thrombocytopenia from temozolomide and radiation in newly diagnosed adults with high-grade gliomas. Neuro-Oncology.

[14] Intile JL, Rassnick KM, Bailey DB, Al-Sarraf R, Chretin JD, Balkman CE (2009). Evaluation of dexamethasone as a chemoprotectant for CCNU-induced bone marrow suppression in dogs. Vet Comp Oncol.

[15] Jakacki RI, Jamison C, Mathews VP, Heilman DK, Dropcho E, Cornetta K (1998). Dose-intensification of procarbazine, CCNU (lomustine), vincristine (PCV) with peripheral blood stem cell support in young patients with gliomas. Med Pediatr Oncol.

[16] Lalatsa A, Lee V, Malkinson JP, Zloh M, Schätzlein AG, Uchegbu IF (2012). A prodrug nanoparticle approach for the oral delivery of a hydrophilic peptide, leucine(5)-enkephalin, to the brain. Mol Pharmaceut.

[17] Chooi KW, Simao Carlos MI, Soundararajan R, Gaisford S, Arifin N, Schätzlein AG (2014). Physical characterisation and long-term stability studies on quaternary ammonium palmitoyl glycol chitosan (GCPQ)- a new drug delivery polymer. J Pharm Sci.

[18] Siew A, Le H, Thiovolet M, Gellert P, Schätzlein A, Uchegbu I (2012). Enhanced oral absorption of hydrophobic and hydrophilic drugs using quaternary ammonium palmitoyl glycol chitosan nanoparticles. Mol Pharmaceut.

[19] Lalatsa A, Garrett N, Moger J, Schätzlein AG, Davis C, Uchegbu IF (2012). Delivery of peptides to the blood and brain after oral uptake of quaternary ammonium palmitoyl glycol chitosan nanoparticles. Mol Pharm.

[20] Moger J, Garrett NL, Begley D, Mihoreanu L, Lalatsa A, Lozano M (2012). Imaging cortical vasculature with stimulated Raman scattering and two photon photothermal lensing microscopy. J Raman Spectroscop.

[21] Fernando LP, Kandel PK, Yu J, McNeill J, Ackroyd PC, Christensen KA (2010). Mechanism of cellular uptake of highly fluorescent conjugated polymer nanoparticles. Biomacromolecules.

[22] Kim JA, Aberg C, Salvati A, Dawson KA (2012). Role of cell cycle on the cellular uptake and dilution of nanoparticles in a cell population. Nat Nanotechnol.

[23] Zumbusch A, Holtom GR, Xie XS (1999). Three-dimensional vibrational imaging by coherent anti-Stokes Raman scattering. Phys Rev Lett.

[24] Garrett NL, Lalatsa A, Begley D, Mihoreanu L, Uchegbu IF, Schätzlein AG (2012). Label-free imaging of polymeric nanomedicines using coherent anti-stokes Raman scattering microscopy. J Raman Spectrosc.

[25] Kastrissios H, Chao NJ, Blaschke TF (1996). Pharmacokinetics of high-dose oral CCNU in bone marrow transplant patients. Cancer Chemother Pharmacol.

[26] Allen TM, Hansen C (1991). Pharmacokinetics of stealth versus conventional liposomes: effect of dose. Biochim Biophys Acta.

[27] Klibanov AL, Maruyama K, Torchilin VP, Huang L (1990). Amphipathic polyethyleneglycols effectively prolong the circulation time of liposomes. FEBS Lett.

[28] Jakacki RI, Yates A, Blaney SM, Zhou T, Timmerman R, Ingle AM (2008). A phase I trial of temozolomide and lomustine in newly diagnosed high-grade gliomas of childhood. Neuro-Oncology.

[29] Franceschi E, Stupp R, van den Bent MJ, van Herpen C, Laigle Donadey F, Gorlia T (2012). EORTC 26083 phase I/II trial of dasatinib in combination with CCNU in patients with recurrent glioblastoma. Neuro-Oncology.

[30] Buyukcelik A, Akbulut H, Yalcin B, Ozdemir F, Icli F (2004). Overdose of lomustine: report of two cases. Tumori.

[31] Kristal O, Rassnick KM, Gliatto JM, Northrup NC, Chretin JD, Morrison-Collister K (2004). Hepatotoxicity associated with CCNU (lomustine) chemotherapy in dogs. J Vet Intern Med.

[32] Billet HH, Walker HK, Hall WD, Hurst JW (1990). Hemoglobin and hematocrit. Clinical methods. the history, physical and laboratory examinations.

[33] Davies LC, Jenkins SJ, Allen JE, Taylor PR (2013). Tissue-resident macrophages. Nat Immunol.

[34] Gregoriadis G, Ryman BE (1972). Fate of protein-containing liposomes injected into rats. an approach to the treatment of storage diseases. Eur J Biochem.

[35] Lalatsa A, Schätzlein AG, Garrett NL, Moger J, Briggs M, Godfrey L (2015). Chitosan amphiphile coating of peptide nanofibres reduces liver uptake and delivers the peptide to the brain on intravenous administration. J Controlled Release.

[36] Glas M, Happold C, Rieger J, Wiewrodt D, Bahr O, Steinbach JP (2009). Long-term survival of patients with glioblastoma treated with radiotherapy and lomustine plus temozolomide. J Clin Oncol.

[37] Lyman GH, Kuderer N, Greene J, Balducci L (1998). The economics of febrile neutropenia: implications for the use of colony-stimulating factors. Eur J Cancer.

[38] Nathan DG (1988). Hematologic diseases.

[39] Harker LA (1977). The kinetics of platelet production and destruction in man. Clin Haematol.

[40] Bolliger AP, Everds N, Hedrich H (2012). Haematology of the mouse. The laboratory mouse.

[41] Spivak JL (1984). Normal hematopoiesis.

[42] Hall JE (2016). Red blood cells, anemia, and polycythemia. Guyton and Hall textbook of medical physiology.

